# Parental Histone Recycling During Chromatin Replication

**DOI:** 10.3390/biom16010013

**Published:** 2025-12-20

**Authors:** Xin Bi

**Affiliations:** Department of Biology, University of Rochester, Rochester, NY 14627, USA; xin.bi@rochester.edu

**Keywords:** chromatin, DNA replication, epigenetic inheritance, histone chaperone, lagging strand, leading strand, nucleosome, parental histone recycling, replisome

## Abstract

The past decade has seen significant advancement in our understanding of DNA replication-coupled chromatin assembly, especially parental histone recycling that is essential for epigenetic inheritance. Leading strand-specific and lagging strand-specific pathways have been found to promote the transfer of parental histones H3-H4 to nascent DNA. It is now clear that the replisome initially characterized as the machinery that carries out the duplication of genomic DNA is also responsible for parental histone recycling. A series of replisome components including CMG (Cdc45-MCM-GINS) replicative helicase, DNA polymerases Polε, Polδ, Polα-primase, and FPC (Fork Protection Complex) that promote parental histone recycling exhibit histone-binding activities. Structural analyses of native and reconstituted replisomes, together with AlphaFold modeling of histone (H3-H4)_2_ tetramer binding by replisome components, provided a framework for understanding the molecular mechanisms of parental histone recycling. A working model has emerged in which the mobile histone chaperone FACT (Facilitates Chromatin Transcription) binds parental histone (H3-H4)_2_ tetramer or (H3-H4)_2_-(H2A-H2B) hexamer on the front of the replication fork, and escorts it across the replisome to the daughter strands in the wake of the replication fork. In this model, parental histones transiently associate with the histone-binding modules in the replisome as steppingstones during their movement. Future studies are needed to elucidate the spatiotemporal coordination of the functions of replisome factors in parental histone transfer.

## 1. Introduction

**Figure 1:**
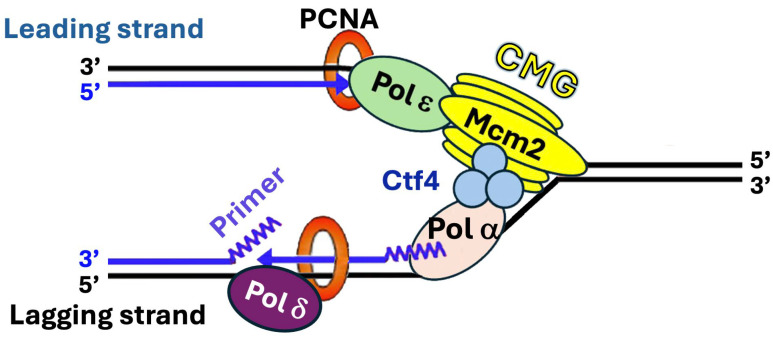
Diagram of a eukaryotic replisome. CMG, Polα, Polδ, Polε, Ctf4, and PCNA of the *S. cerevisiae* replisome are shown. Other replisome components including Tof1, Csm3, Mrc1, and FACT are not shown for clarity. Note that the eukaryotic replisome is highly conserved from yeast to mammals [1]. Template and nascent strands are shown as black and blue lines, respectively. See the text for descriptions.

DNA replication is the precise duplication of the genome that ensures the faithful transmission of genetic information across generations. It is a highly orchestrated process carried out by the replisome, a multiprotein complex consisting of a DNA helicase for separating the two strands of double-stranded DNA (dsDNA), a primase for making primers for replication, DNA polymerases for synthesizing new strands of DNA, and accessory factors that help organize the replication machinery on DNA template and ensure high processivity of DNA synthesis [1]. In eukaryotes, DNA replication starts with the unwinding of DNA double helix by the Cdc45-MCM-GINS (CMG) replicative helicase into two single-stranded DNA (ssDNA) strands, creating the replication fork at which the replisome assembles [1] (Figure 1). Two distinct polymerases ε and δ (Polε and Polδ) are responsible for the bulk of leading strand and lagging strand replication, respectively. There is evidence that Polδ also plays roles in the initiation and termination of leading strand replication [2]. Polε or Polδ extends a primer synthesized by the Polα-primase complex. CMG at the front of the replisome tracks along the leading strand [1] (Figure 1). It interacts with a series of factors including Facilitates Chromatin Transcription (FACT) chromatin remodeling complex (Spt16-Pob3) and Ctf4 homotrimer to promote the movement of replication fork along the DNA template [1,3] (Figure 1). Polα-primase binds directly to the leading edge of the CMG via a complex interaction network [4]. CMG also binds Polε and helps stabilize Polε on the leading strand, which contributes to the processivity of Polε in DNA synthesis [1] (Figure 1). The proliferating cell nuclear antigen (PCNA) is a key replisome component that orchestrates multiple activities at the replication fork by serving as a platform for the recruitment of key replication factors [5] (Figure 1). PCNA, a homotrimer of the Pol30 protein, encircles DNA to form a DNA sliding clamp [5]. PCNA binds both Polε and Polδ and serves as a processivity factor for them [5] (Figure 1). Three replisome components, Tof1, Csm3, and Mrc1, interact to form the Fork Protection Complex (FPC) that acts on the front of the replisome to promote stable replication and replication-associated activities such as checkpoint response to DNA lesions [6].

The eukaryotic genome is packaged into chromatin that is critical for the maintenance and function of the genome [7]. Chromatin regulates DNA transactions including gene transcription, DNA replication, recombination, and repair. The basic repeating unit of chromatin is the nucleosome composed of ~147 base pairs of DNA wrapping around a protein octamer made of two copies of each core histone, H2A, H2B, H3, and H4 [8]. The histone octamer in the nucleosome assumes a characteristic tripartite structure consisting of a histone (H3-H4)_2_ tetramer flanked by two (H2A-H2B) dimers. Nucleosome formation is achieved by the deposition of the histone (H3-H4)_2_ tetramer onto DNA, followed by the addition of two (H2A-H2B) dimers [9]. Histones are subject to extensive post translational modifications (PTMs) such as lysine acetylation, methylation, and ubiquitination that modulate the structure and function of nucleosome/chromatin [10]. The genome is organized into structurally and functionally distinct chromatin domains with different patterns of histone PTMs and different degrees of compaction that collectively determine chromatin-dependent epigenetic states. Although nucleosome formation involves strong interactions between negatively charged DNA with positively charged histones, the nucleosome structure is dynamic, allowing regulated DNA access. Nucleosomal (H2A-H2B) dimers can readily exchange with newly synthesized H2A-H2B during the cell cycle [11]. Histone (H3-H4)_2_ tetramers are believed to be the primary carriers of epigenetic modifications.

DNA replication in eukaryotes is closely coupled with the disassembly and reassembly of chromatin to ensure the duplication and inheritance of both genetic and epigenetic information. Nucleosomes formed on nascent DNA contain half parental (“old”) histones and half newly synthesized (“new”) histones [12]. Notably, the (H3-H4)_2_ tetramer displaced from a parental nucleosome is transferred as an intact unit to nascent DNA, and old and new (H3-H4)_2_ tetramers are randomly distributed onto leading and lagging strands of DNA replication forks [11,13]. The new histones in newly assembled chromatin will adopt the PTMs of the old histones through a “read-write” mechanism [14]. The question of how old and new histones are deposited onto nascent DNA during DNA replication is of great importance for understanding chromatin-based epigenetic inheritance.

**Figure 2:**
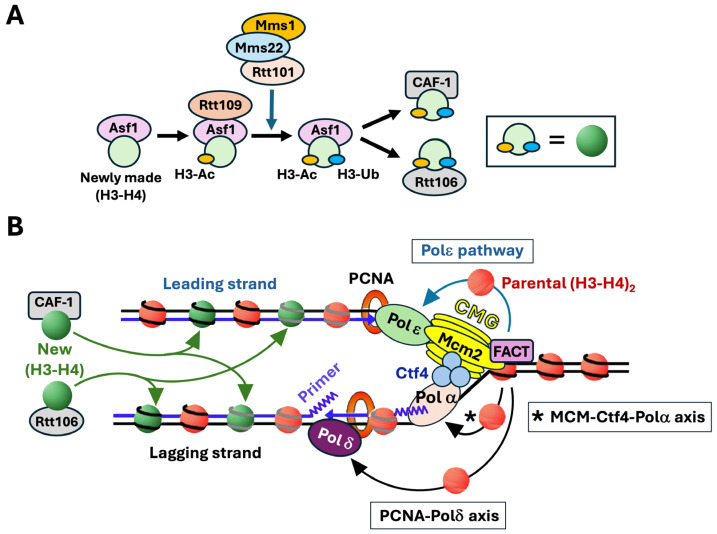
Replication-coupled chromatin assembly. (**A**). Posttranslational modification of newly synthesized histones H3-H4 for their deposition on nascent DNA by chromatin assembly factors. Newly synthesized histone (H3-H4) dimer is chaperoned by Asf1. Rtt109 binds Asf1 and acetylates histone H3-K56 (H3-Ac). Rtt101/Mms1/Mms22 then ubiquitinates H3-K121, -K122, and -K125 (H3-Ub). H3-Ac and H3-Ub facilitate the handover of (H3-H4) dimer from Asf1 to CAF-1 and Rtt106 chromatin assembly factors. (**B**) Deposition of newly synthesized and parental histones H3-H4 on daughter strands at the DNA replication fork. Left, newly synthesized histones H3-H4 marked by H3-Ac and H3-Ub (green circles) are deposited by CAF-1 or Rtt106 to both leading and lagging strands. Right, parental histone (H3-H4)_2_ tetramers (red circles) are transferred to the leading strand via the Polε pathway, and to the lagging strand through the MCM-Ctf4-Polα axis and PCNA-Polδ axis. Replisome components CMG, Polα, Polδ, Polε, Ctf4, PCNA, and FACT are shown. See the text for more descriptions.

Newly synthesized histones H3-H4 are bound by histone chaperones and are subject to specific modifications before their deposition onto nascent DNA [12,15] (Figure 2A). In this process, newly synthesized histone (H3-H4) dimer associates with histone chaperone Asf1 and is acetylated at lysine 56 of histone H3 (H3-K56Ac) by histone acetylase Rtt109, and then ubiquitinated at H3-K121, -K122, and -K125 by the Rtt101/Mms1/Mms22 ubiquitinase [12,16,17] (Figure 2A). Histone H3 acetylation and ubiquitination facilitate the transfer of (H3-H4) dimer from Asf1 to Chromatin Assembly Factor-1 (CAF-1) complex and Rtt106 for deposition on both daughter strands (Figure 2). FACT also contributes to new histone deposition during DNA replication redundantly and in corporation with CAF-1 and Rtt106 [18].

The transfer of parental histone (H3-H4)_2_ tetramer to nascent DNA during DNA replication is promoted by a group of replisome components including CMG, DNA polymerases, FPC, as well as FACT, all of which have histone chaperone activities [19]. Recent studies have revealed leading and lagging strand-specific pathways of parental histone recycling and structural and molecular insights into the stepwise transmission of parental histones from the front of the replisome to daughter strands in the wake of the replication fork. These exciting findings are the focus of this review.

## 2. Discovery of Strand-Specific Pathways for the Recycling of Parental Histones H3-H4 During DNA Replication

There was evidence suggesting the existence of an active mechanism of escorting parental histones H3-H4 released from the nucleosome in front of the replication fork to nascent DNA strands in the wake of the fork which is separable from the deposition of newly synthesized histones H3-H4 [20,21]. Delineation of this mechanism required the identification of the proteins involved especially those that directly interact with parental histones H3-H4. The finding of a histone binding domain (HBD) in the Mcm2 subunit of CMG that binds histone H3-H4 provided an early clue [22,23,24]. Notably, HBD of Mcm2 is dispensable for the DNA helicase activity of CMG but is important for transcriptional silencing in yeast [25]. As such, in addition to being a DNA helicase, CMG is a chaperone of H3-H4 that contributes to the formation or maintenance of heterochromatin, possibly by contributing to parental histone recycling.

A breakthrough came in 2018 when a role of Mcm2 in facilitating the transfer of parental histones H3-H4 to lagging strand and a role of Polε in promoting parental H3-H4 transfer to leading strand were revealed [26,27,28]. The key to the success of these studies was the use of the eSPAN method (enrichment and sequencing of protein-associated nascent DNA) or a similar method SCAR-seq (sister chromatids after replication by DNA sequencing) that allows the detection of leading strand- or lagging strand-specific association of a protein at the replication fork [27,29]. The eSPAN method enables the tracking of parental histones carrying characteristic PTMs such as histone H3-K4me3 and newly made histones carrying the characteristic PTM histone H3-K56ac on daughter strands. Using eSPAN, Gan et al. showed that mutating HBD of yeast Mcm2 led to a strong leading strand bias for the association of parental (H3-H4)_2_ with leading strand and a concomitant strong lagging strand bias for the association of new (H3-H4)_2_ with lagging strand, which is due to the impairment of the transfer of parental (H3-H4)_2_ to lagging strands [26]. Using SCAR-seq, Petryk et al. made a similar finding in mouse embryonic stem cells (ESCs) [27]. As such, the Mcm2 subunit of CMG, via its (H3-H4)_2_ binding activity, acts to direct the transfer of parental (H3-H4)_2_ to the lagging strand, a function that is conserved from yeast to mammalian cells.

CMG is connected to Polα-primase on the lagging strand via Ctf4 that binds both the Sld5 subunit of CMG and the Pol1 subunit of Polα-primase [2,30,31] (Figure 2B). Gan et al. further showed that disruption of CMG-Polα-primase connection by mutating Ctf4 or Pol1 resulted in a leading strand bias of parental (H3-H4)_2_ association similar to mutating Mcm2-HBD [26]. It was later found that Pol1 binds H3-H4 and its histone binding motif is important for parental H3-H4 transfer to lagging strands in both yeast and mouse ESCs [32]. These findings revealed an Mcm2-Ctf4-Polα axis that promotes the transfer of parental (H3-H4)_2_ displaced from the nucleosome in front of the replication fork to the lagging strand (Figure 2B).

Yu et al. found that deletion of Dpb3 or Dpb4, two non-essential subunits of Polε, resulted in a marked preference for the association of parental (H3-H4)_2_ with lagging strands and a loss of transcriptional silencing in yeast [28]. Importantly, Dpb3-Dpb4 complex was found to be a histone H3-H4 chaperone [28,33]. These results indicate that Polε, via its Dpb3-Dpb4, helps to direct the transfer of parental (H3-H4)_2_ to leading strands (Figure 2B). Li et al. later found evidence that the functional homologs of yeast Dpb3 and Dpb4 in mouse ESCs are also required for transferring parental H3-H4 to leading strands [32].

Like the leading strand DNA polymerase Polε, the lagging strand DNA polymerase Polδ was recently also found to be a histone H3-H4 chaperone that facilitates parental histone recycling [34,35,36]. It was further found that it is Pol32, the accessory subunit of Polδ, that binds histones H3-H4 and facilities their transfer to lagging strands [34,35,36]. Tian et al. found that Pol32 binds Mcm2, which is independent of Ctf4 and is therefore different from the Ctf4-bridged Polα-Mcm2 interaction [34]. Pol32-Mcm2 interaction is markedly enhanced by the presence of histones H3-H4, and Pol32 interaction with H3-H4 is dependent on Mcm2 [34]. These results led to the notion that Polδ receives H3-H4 from Mcm2 of CMG before transferring it to lagging strands [34] (Figure 2B).

The processivity and catalytic activity of Polδ in DNA replication is critically dependent on its interaction with PCNA [5]. Serra-Cardona et al. examined whether the role of Polδ in parental histone recycling is also dependent on its interaction with PCNA [35]. PCNA interacts directly with a host of proteins involved in various processes such as DNA replication and chromatin assembly [5]. As a homotrimer of the Pol30 protein, PCNA is believed to be able to bind multiple proteins at the same time. Many PCNA-interacting proteins contain conserved motifs called PIP (PCNA-interacting protein) boxes that mediate their binding to PCNA. All three components of Polδ, Pol3, Pol31, and Pol32, process PIP boxes [35]. Pol30 mutations that affect PCNA interaction with specific proteins have been characterized. The *pol30-79* mutation is known to disrupt PCNA-Polδ interaction [37]. Serra-Cardona et al. found evidence that *pol30-79* impairs parental histone H3-H4 transfer to lagging strand [35]. Moreover, mutating the PIP box of Pol3 and to a lesser extent that of Pol32 also impairs parental H3-H4 transmission to lagging strands [35]. These results indicates that PCNA plays a role in parental histone transfer to lagging strands by interacting with Polδ (mainly its Pol3 subunit) [35] (Figure 2B).

## 3. Role of Mrc1 in Coordinating Symmetric Parental Histone Transfer During DNA Replication

Another significant recent progress in research on DNA replication-coupled chromatin assembly is the identification of Mrc1 as a histone H3-H4 chaperone that promotes parental histone recycling [38]. Mrc1 as a component of FPC is known to serve as a mediator of DNA replication checkpoint [39]. An early study found that Mrc1 also plays a role in efficient transcriptional silencing in the budding yeast [40]. Similarly, fission yeast Mrc1 was recently shown to be required for the maintenance of heterochromatin, which is separable from its role in replication checkpoint signaling [41,42,43].

Using the *AlphaFold2*-multimer protein complex prediction program, Yu et al. and Charlton et al. simultaneously identified a potential interface between a region of Mrc1 and (H3-H4)_2_ tetramer [41,42]. Direct interaction between Mrc1 and (H3-H4)_2_ tetramer was then confirmed by in vitro protein–protein interaction analyses [41,42]. Alanine mutagenesis of key residues in the predicted (H3-H4)_2_ binding domain of Mrc1 impairs its interaction with H3-H4 and disrupts heterochromatin maintenance [41,42]. One mutation, *mrc1-3A* (M755A, F756A, L774A), was shown to reduce the level of parental histones H3-H4 transmitted to both leading and lagging strands to roughly the same extent, suggesting a role of Mrc1 in the transfer of H3-H4 to both strands [41]. Interestingly, certain other mutations of Mrc1 were found to cause either a leading strand or lagging strand bias in the transfer parental histones H3-H4 [42,43]. It is possible that these mutations affect *separate functions* of Mrc1 in the recycling of parental histones to the leading and lagging strands [42]. Consistent with this notion, there is evidence suggesting that Mrc1 collaborates with Mcm2 to promote parental histones H3-H4 transfer to lagging strands [42,43] while facilitating parental histones H3-H4 transfer to leading strands redundantly with Polε [41]. These results collectively suggest that Mrc1 regulates the distribution of parental histones to strand-specific pathways via its HBD, while coordinating the functions of other replisome components to ensure symmetry in parental histone transfer through its other domains [41,42].

## 4. FACT Participates in Parental Histone Transfer to Both Leading and Lagging Strands

The FACT complex is a multifunctional histone chaperone involved in chromatin remodeling during DNA transactions including transcription and replication. FACT was initially identified as a histone chaperone that facilitates transcription through chromatin by removing H2A-H2B dimers from nucleosomes [44,45,46,47,48]. It binds both H2A-H2B and H3-H4 and has both nucleosome disassembly and assembly activities [47,49,50,51]. FACT promotes efficient replication through chromatin by cooperating with CMG to disrupt nucleosomes and unwind DNA in front of the replication fork [3,20,52,53]. Moreover, FACT also functions in the deposition of newly synthesized histones on nascent strands during DNA replication [18,54]. Recently, FACT was found to also play a role in parental histone transfer to lagging and leading strands [41,55,56]. Depletion of either the Spt16 or Pob3 subunit of FACT results in a significant decrease in prenatal histone density on both leading and lagging strands [55,56]. FACT complex acts in concert with Mcm2 of CMG and Dpb3-Dpb4 of Polε for parental histone recycling [55,56] (Figure 2B). The N-terminal domain of Spt16 interacts with Mcm2 to facilitate lagging strand-specific parental histone transfer [55]. FACT also interacts with three other replisome components Mrc1, Tof1, and Pol1 [41,53]. Therefore, FACT likely cooperates with multiple replisome components to promote parental histone recycling.

## 5. Molecular Mechanisms of Parental Histone Recycling Mediated by the Replisome

**Figure 3:**
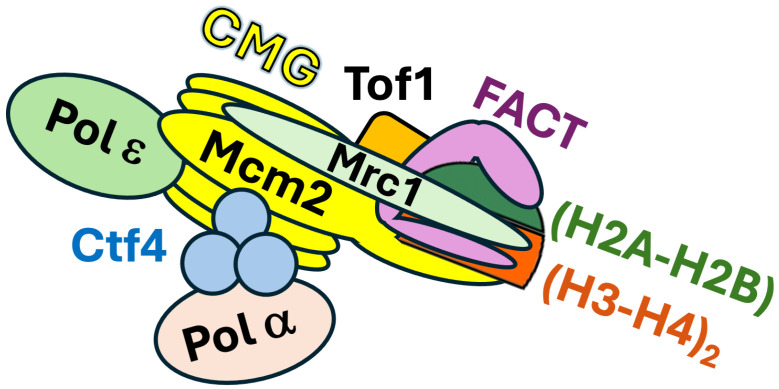
Schematic diagram of a replisome associated with FACT and histones. The diagram is based on the cryo-EM structure of a native replisome [57]. An (H3-H4)_2_-(H2A-H2B) hexamer is cochaproned by FACT and Mcm2 of CMG at the front (right side) of the replisome. Note Mrc1 is not readily “visible” in the cryo-EM structure of native replisome [57]. Based on AlphaFold2-multimer modeling and biochemical studies, Mrc1 is shown to bind both CMG and (H3-H4)_2_ in the hexamer [41,42], which awaits validation by future structural analysis.

The recent findings described above identified a series of DNA replication factors involved in parenteral histone recycling but did not address exactly how they carry out the chain of events of nucleosome disruption in front of the fork and the transmission of evicted parental histones to daughter strands in the context of a native replisome at a replication fork. The complexity and dynamic nature of the machinery of chromatin replication make it challenging to address this question. Li et al. sought to address this question by using cryo-electron microscopy (cryo-EM) to capture snapshots of the structure of replisome in the action of transmitting parental histones to nascent DNA at the replication fork [57]. To this end, they affinity purified chromatin-bound replisomes from budding yeast cells synchronized in early S phase by hydroxyurea (HU) treatment. The isolated complexes consisted of CMG, Polε, Ctf4, Tof1, Csm3, Mrc1, histones, and FACT in high abundance, as well as Polα and other factors in relatively low abundance (Figure 3). Notably, histones in the complexes carry the H3-K4me3 marker characteristic of parental histone H3, but not the H3-K56ac marker specific for newly synthesized H3 [57]. Cryo-EM analysis revealed a high-resolution structure of a native replisome associated with FACT and parental histones that provides molecular details of an early step of parental histone transfer. In this structure, the Spt16 subunit of FACT binds to a histone hexamer (histone octamer missing one (H2A-H2B) dimer) at the front end of the replisome (Figure 3). The vacant (H2A-H2B) dimer site is occupied by the HBD of Mcm2 such that Mcm2 and FACT bind together (or cochaperone) the (H3-H4)_2_ tetramer part of the evicted histone hexamer (Figure 3). Csm3/Tof1 are positioned ahead of CMG at the front of the replisome where they grip duplex DNA [57], which stabilizes the whole complex [58] (Figure 3). Notably, the evicted histone hexamer cochaperoned by FACT and Mcm2 is associated with one part (the head region) of Tof1, whereas parental DNA binds a different part (the body) of Tof1 [57]. As such, Tof1 is believed to help separate parental histones from parental DNA [57]. Mcm2 interacts with Tof1, and disruption of this interaction by Tof1 or Mcm2 mutations leads to a leading strand bias of parental histone distribution on nascent DNA strands [57]. These results indicate that Mcm2-Tof1 coupling plays a role in parental histone transfer to lagging strands.

The interactions among FACT, Mcm2, Tof1, and evicted parental histone hexamer at a key step of chromatin replication revealed by cryo-EM (Figure 3) indicates that the histone octamer from the parental nucleosome in front of replication fork is first split into an (H2A-H2B)-(H3-H4)_2_ hexamer and an (H2A-H2B) dimer during nucleosome disassembly. The evicted histone hexamer is cochaperoned by Spt16 and Mcm2 and directed to the replisome front over the head region of Tof1 (Figure 3). Whether the histone hexamer is further split into one (H3-H4)_2_ tetramer and one H2A-H2B dimer before the (H3-H4)_2_ tetramer is transferred to daughter strands, or the hexamer is recycled as an intact unit, has yet to be resolved.

It is noteworthy that the replisomes examined by Li et al. was isolated from cells synchronized in early S phase by HU that stalls the progression DNA replication forks [57]. HU has long been known to inhibit ribonucleotide reductase involved in the synthesis of deoxynucleotide triphosphates (dNTPs), thereby impairing DNA synthesis and stalling DNA replication [59]. HU was recently found to also induce reactive oxygen species (ROS) generation that inhibits activities of Polα, Polδ, and Polε [60]. Although replisomes remain associated with replication forks during replication stress [61], they may be structurally different from replisomes at unperturbed forks. For example, replication stress may induce the decoupling of DNA helicase and polymerase, and ROS induced by HU disrupts the association of DNA polymerases with template DNA [60,62]. Therefore, it is formally possible that the cryo-EM structure of the replisome reported by Li et al. may not completely reflect the configuration of unperturbed replisomes during normal DNA replication. That said, the cryo-EM structure is generally in agreement with cryo-EM structures of several reconstituted replication complexes previously reported [58,63,64,65].

In the cryo-EM image of native replisome, signals corresponding to Mrc1 are not obvious except for a small region that is connected to Tof1 [57]. Mrc1 was also not resolved in the cryo-EM structures of reconstituted replication complexes [58,61]. Using crosslinking mass spectrometry Baretić et al. showed that Mrc1 makes contacts with Tof1 and Ctf4, as well as Mcm2, Mcm6, and Cdc45 across one side of CMG [58]. This is consistent with, and complements, previously demonstrated interactions of Mrc1 with Csm3-Tof1, Mcm6, and Polε [66,67,68,69] and recent AlphaFold2-multimer predicted interactions of Mrc1 with Mcm2 and Cdc45 [41,43]. That Mrc1 interacts with both factors (Csm3/Tof1) at the front of the replisome and those behind CMG (Mcm6 and Polε) [70,71] suggests that it stretches from the front to the rear of the replisome (58) (Figure 3). Moreover, AlphaFold2-multimer modeling of Mrc1-(H3-H4)_2_ complex revealed that Mrc1 makes multiple contacts with histones H3 and H4 to form a brace around (H3-H4)_2_ (Figure 3). Notably, this brace is compactable with Mcm2 binding to (H3-H4)_2_, indicating that Mrc1 and Mcm2 can collaborate to cochaperone (H3-H4)_2_ (Figure 3) [41,42]. As such, Mrc1 may bind histones (H3-H4)_2_ alone or together with Mcm2 to direct their “movement” from the front to the rear of the replisome during parental histone recycling.

In the cryo-EM structure of native replisome, Polε exists in two major conformations [57]. Polε is stably engaged with the motor domain of CMG in one conformation, while being highly flexible in the other conformation with the N-terminal domain (NTD) of its Dpb2 subunit attached to CMG [57]. It was proposed that Polε participates in parental histone recycling by alternating between these two states. Upon adopting the flexible conformation, Polε may flip over CMG to the front of the replication fork, thereby allowing its Dpb3-Dpb4 component to capture (H3-H4)_2_ tetramer chaperoned by FACT [57]. There is genetic evidence suggesting that Mrc1 and Polε function together in parental histone transfer to leading strands [41]. Consistent with this notion, Mrc1 is known to interact with Pol2, the catalytic subunit of Polε [66].

Although the evicted (H3-H4)_2_ tetramer in the native replisome is cochaperoned by Spt16 of FACT and Mcm2 of CMG, the surface of one (H3–H4) dimer facing Ctf4 is partially exposed [57]. This surface is predicted by a cryo-EM structure of reconstituted replisome to be accessible to the N-terminal domain of Pol1 [4,41,57]. Therefore, it is likely that Polα is directed by Ctf4 to a strategic location in the replisome to receive FACT-(H3-H4)_2_ from CMG for deposition on lagging strands. Recent AlphaFold2-multimer modeling analyses suggest that Mrc1 facilitates the association of Polα with Mcm2 and the positioning of the histone binding domain of Mcm2 near Polα, thereby possibly assisting the Mcm2-Ctf4-Polα axis of parental histone transfer to lagging strands [26,43].

Polδ was absent from recently reported cryo-EM structures of native or reconstituted replisomes [57,58]. As such, there is a lack of structural insights into how Polδ may receive parental histones H3-H4 from upstream chaperones in the replisome for deposition on lagging strands. Using single-molecule imaging, Lewis et al. found evidence for the tethering of Polδ to a stable part of the replisome, which is dependent of Pol32 [72]. Polδ is also known to interact with Polα-primase (via Pol32-Pol1 interaction) [73,]. Notably, Polδ-Polα-primase interaction is enhanced by H3-H4 (34), raising the possibility that Polδ and Polα-primase cochaperone parental (H3-H4)_2_ at a stage of its transfer to the lagging strand. It is possible that parental histones H3-H4 pass from Polα to Polδ during their transfer to the lagging strand [35]. On the other hand, Tian et al. found evidence suggesting that Pol1 subunit of Polα may receive (H3-H4)_2_ from Pol32 of Polδ during parental histone transfer [34]. Further investigation is needed to clarify the functional relationship between Polδ and Polα in chaperoning parental histones during their transfer to lagging strands.

**Figure 4:**
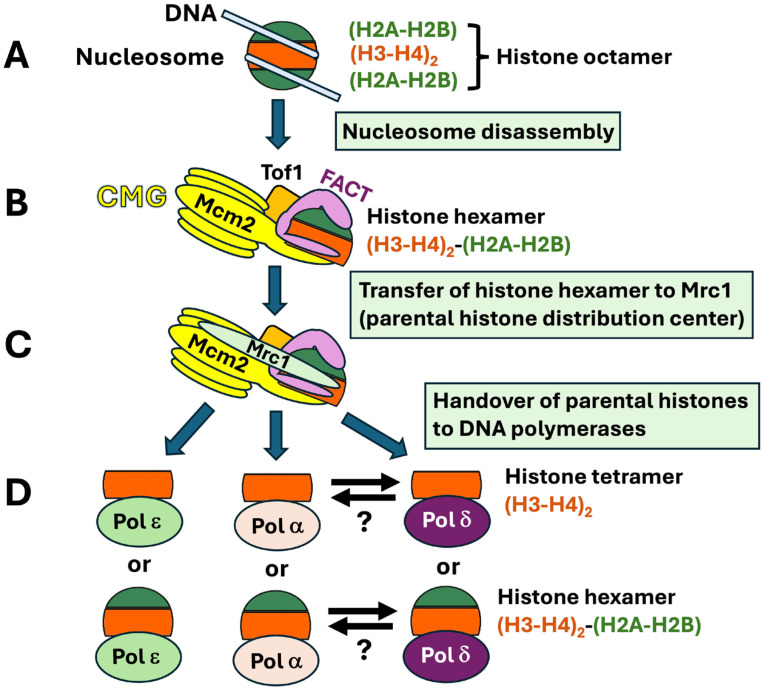
Model for stepwise transfer of parental histones to DNA polymerases. The nucleosome (**A**) in front of the replication fork is disrupted by the combined action of CMG and FACT, and a (H2A-H2B) dimer is removed from the histone octamer. The remaining (H3-H4)_2_-(H2A-H2B) hexamer is cochaperoned by Mcm2 and FACT (**B**). The (H3-H4)_2_-(H2A-H2B) hexamer, or (H3-H4)_2_ tetramer, chaperoned by FACT, is transferred to Tof1-Mrc1 as the parental histone distribution center (**C**). The (H3-H4)_2_-(H2A-H2B) hexamer, or (H3-H4)_2_ tetramer, is then handed over to Polε for deposition on the leading strand and to Polα or Polδ for deposition on the lagging strand (**D**). It is possible that the histone tetramer or hexamer is still chaperoned by FACT in this process. Note that the histone tetramer or hexamer might “hop” from Polα to Polδ, or vice versa, as indicated by the antiparallel arrows.

Taken together, the recently characterized cryo-EM structures of native and reconstituted replisomes, the AlphaFold2-multimer modeling predicted interactions among Mrc1, Mcm2, and (H3-H4)_2_ and a large body of genetic and biochemical evidence provide the basis for a step-by-step model of parental histone recycling [26,27,28,32,33,34,35,36,41,42,43,57,58,74]. In this model, *CMG* destabilizes the nucleosome in front of the replication fork and FACT binds to the partially unraveled nucleosome (Figure 4A,B). CMG and FACT split the histone octamer into an (H2A–H2B) dimer and a (H2A-H2B)-(H3-H4)_2_ hexamer that is cochaperoned by Spt16 of FACT and Mcm2 of CMG (Figure 4B). (The histone hexamer may or may not be further split into an H3-H4 tetramer and a H2A-H2B dimer.) The histone tetramer (or hexamer) is then directed to Tof1-Mrc1 that serves as a parental histone distribution center (Figure 4C). Subsequently, the histone tetramer is handed over to Polε for deposition onto the nascent leading strand, or Polα and Polδ for deposition onto the lagging strand (Figure 4D).

## 6. Conclusions and Outlook

Considerable progress has been made recently in delineating the molecular mechanism of parental histone recycling. The replisome that carries out the replication of genomic DNA is also responsible for parental histone recycling. Notably, a series of replisome factors (Mcm2, Pol32, Dpb3-Dpb4, Pol1, and Mrc1) that promote parental histone transfer have been shown to be histone chaperones (Table 1). These histone-binding interfaces on the replisome are believed to form a network of steppingstones that guides the passage of parental histone (H3-H4)_2_ tetramers from the front of the replication fork to daughter strands in the wake of the fork. In this model, the histone H3-H4 tetramer, or (H3-H4)_2_-(H2A-H2B) hexamer, evicted from the nucleosome in front of the replication fork is escorted by the mobile/dynamic chaperone FACT (and ASF1 in mammalian cells) during their transmission across the replisome. At each intermediate step, the (H3-H4)_2_ tetramer is effectively cochaperoned by FACT and a steppingstone chaperone on the replisome. In support of the model, FACT and Mcm2 are found to cochaperone histone (H3-H4)_2_ at the front edge of the replication fork in the recently reported cyo-EM structure of native replisomes. Moreover, cochaperoning of (H3-H4)_2_ by FACT and Mrc1 and that by FACT and Pol1 have been predicted by *AlphaFold2-multimer modeling.*

Given the complexity of the replisome and limited structural information of it, our understanding of how the many replisome components coordinate to promote symmetric/equal distribution of parental histones on both leading and lagging strands of DNA replication is far from complete. The proposed sequence of events of parental histone (H3-H4)_2_ transfer across the replisome await characterization. More structural analyses of native replisomes at different stages of prenatal histone recycling are needed to elucidate the proposed intermediates of parental histone transfer that involve the cochaperoning of (H3-H4)_2_ by the mobile FACT chaperone and histone-binding replisome components. Examination of the structures of replisomes individually mutated for factors required for parental histone transfer may reveal the functions of these factors in the context of the replisome at the replication fork. The existence of a native replisome associated with FACT and a parental histone hexamer raises the possibility that the (H3-H4)_2_-(H2A-H2B) hexamer is recycled as a unit. However, whether this hexamer loses the H2A-H2B dimer before being deposited on DNA has yet to be clarified. Moreover, how cells coordinate parental histone recycling and new histone deposition to ensure equal distribution of parental and newly synthesized histones onto nascent DNA has yet to be investigated. Addressing these outstanding questions would enhance our understanding of the mechanism of chromatin replication that is critically important for epigenetic inheritance.

## Figures and Tables

**Table 1:** Replisome components as histone chaperones involved in parental histone transfer.

Name	Complex	Histone-Binding	References
Pol1	Polα-primase	H2A-H2B and H3-H4	[32,75]
Pol32	Polδ	H3-H4	[34,35,36]
Dpb3-Dpb4	Polε	H3-H4	[28,33]
Mcm2	CMG	H3-H4	[24,25,42,55]
Spt16	FACT	H2A-H2B and H3-H4	[49,50]
Mrc1	FPC	H3-H4	[41,42]

## Data Availability

No new data were created or analyzed in this study.
